# Draft Genome Sequence of *Escherichia coli* Strain Nissle 1917 (Serovar O6:K5:H1)

**DOI:** 10.1128/genomeA.00047-13

**Published:** 2013-02-28

**Authors:** Brady F. Cress, Robert J. Linhardt, Mattheos A. G. Koffas

**Affiliations:** a Department of Chemical and Biological Engineering, Center for Biotechnology and Interdisciplinary Studies, Rensselaer Polytechnic Institute, Troy, New York, USA; b Department of Chemistry, Chemical Biology, Center for Biotechnology and Interdisciplinary Studies, Rensselaer Polytechnic Institute, Troy, New York, USA; c Department of Biology, Center for Biotechnology and Interdisciplinary Studies, Rensselaer Polytechnic Institute, Troy, New York, USA

## Abstract

We announce the availability of the 5.023-Mbp high-quality draft assembly of the *Escherichia coli* strain Nissle 1917 (serovar O6:K5:H1) genome. Short genomic segments from this important probiotic strain have been available in public databases, but the full genome sequence has remained inaccessible. Thus, high-coverage, whole genome sequencing of *E. coli* Nissle 1917 is presented herein. Reannotation and metabolic reconstruction will enable comparative genomics analysis and model-guided predictions of genetic manipulations leading to increased production of the K5 capsular polysaccharide known as *N*-acetyl heparosan, a precursor to the anticoagulant pharmaceutical heparin.

## GENOME ANNOUNCEMENT

The commensal *Escherichia coli* strain Nissle 1917 is one of the oldest, most well-characterized probiotic agents and has shown promising results in treatment of various intestinal diseases and disorders (1) since its isolation from the feces of a World War I soldier exhibiting unique intestinal fortitude compared to his cohort, all of whom developed infectious diarrhea (2). Assorted genomics studies have been performed on Nissle 1917, including tRNA screening, genomic island sequencing, DNA-DNA hybridization (3), and even low-coverage genomic shotgun sequencing (4). Until now, however, the whole genome sequence has been inaccessible. Serotyping of *E. coli* Nissle 1917 has identified the presence of a K5 antigen, which is known to be composed of *N*-acetyl heparosan (a precursor to the anticoagulant pharmaceutical heparin), a group 2 capsular polysaccharide (CPS) consisting of a repeating [→4) β-d-glucuronic acid (GlcA) (1 → 4) *N*-acetyl-α-d-glucosamine (GlcNAc) (1→]_*n*_ disaccharide unit (5). Under certain growth conditions, *E. coli* Nissle 1917 produces significantly more CPS than *E. coli* K5 (data not shown), making the organism attractive as a production strain for bioengineered heparin.

*Escherichia coli* Nissle 1917 was cultured from a Mutaflor tablet (Ardeypharm, Herdecke, Germany) and plated on Luria-Bertani (LB) agar medium; a single colony was picked and grown in LB medium at 37°C overnight, and the genomic DNA was purified with an Invitrogen PureLink genomic DNA mini kit. Prior to whole-genome sequencing, successful PCR amplification of the genes *kfiA*, *kfiB*, *kfiC*, and *kfiD* in region 2 of the capsular polysaccharide biosynthetic gene cluster confirmed probable isolation of Nissle 1917. The genome was sequenced using the Illumina HiSeq 2000 sequencing system, which produced 110 M paired-end reads of 101 bp with an insert size of 400 bp. Approximately 28 M random reads were assembled with Velvet v1.2.07 (6) at an optimal hash length of 91. The final genome assembly has 51-fold coverage and contains 125 supercontigs composed of 143 contigs (>200 bp in length) with a total size of 5,023,325 bp, an N_50_ contig length of 253,628 nucleotides, and a mean G+C content of 50.5%. All assembly data were deposited in the EMBL nucleotide sequence database.

The draft genome was annotated by the RAST (Rapid Annotation using Subsystem Technology) server (7) using Glimmer3 as a gene caller (8), which predicted 4,846 coding sequences (CDSs) with an average length of 900 bp (3,739 CDSs have functional predictions), 80 tRNA-encoding genes, and 9 rRNA-encoding genes. RAST was also used to construct a draft metabolic model (9) containing 1,179 genes, corresponding to 1,388 reactions with 1,089 metabolites (including 4 gap-filling reactions and an artificial biomass reaction). Comparison of metabolic reconstructions will uncover differential carbohydrate and polysaccharide biosynthetic pathways between Nissle 1917 and K5 while yielding insight on growth conditions leading to maximum CPS production. A comparative genomics analysis currently ongoing in our lab between Nissle 1917 and related uropathogenic *E. coli* (UPEC) strains producing similar capsular polysaccharides will also guide understanding of the role of glycosaminoglycan-like capsules in pathogenesis.

### Nucleotide sequence accession numbers.

The annotated draft genome sequence was deposited in DDBJ/EMBL/GenBank under accession no. CAPM00000000. The version described in this paper is the first version, CAPM01000000.
